# Editorial: Neuroscience of Human Attachment

**DOI:** 10.3389/fnhum.2017.00136

**Published:** 2017-03-24

**Authors:** Anna Buchheim, Carol George, Harald Gündel, Roberto Viviani

**Affiliations:** ^1^Institute of Psychology, University of InnsbruckInnsbruck, Austria; ^2^Department of Psychology, Mills CollegeOakland, CA, USA; ^3^Department of Psychosomatic Medicine and Psychotherapy, Ulm UniversityUlm, Germany

**Keywords:** attachment, attachment measures, human neuroscience, psychophysiology, EEG/ERP, fMRI, oxytocin, genetic markers

The Research Topic “Neuroscience of Human Attachment” includes innovative papers representing a broad spectrum of contemporary approaches to the investigation of biologically based systems that guide cognitive and emotional processes associated with intimate and significant relationships. This spectrum includes studies and theoretical reviews that discuss neurobiological substrates (fMRI, EEG, psychophysiology, endocrine parameters, genetic polymorphisms) using a range of psychometric approaches to attachment assessment [interview like e.g., the Adult Attachment Interview (AAI) (George et al., unpublished manuscript; Main and Goldwyn, unpublished manuscript), free response like e.g., the Adult Attachment Projective Picture System (AAP) (George and West, 2012), self-report questionnaire like e.g., the Relationship Scales Questionnaire].

The first group of papers explored the identification of neural activation response patterns to different relationship-based stimuli presented in an fMRI context. Heckendorf et al. examined the effects of subliminal threatening primes on responses to the presentation of familiar and unfamiliar faces. Their study showed enhanced activity in social cognition areas in the posterior temporal/anterior parietal lobes in response to viewing unfamiliar faces, indicating increased effortful processing. Labek et al. showed the involvement of similar social cognitive cortical areas in response to viewing AAP attachment stimuli as compared with carefully matched control pictures. Interestingly, Davidovic et al. full the same neural system was also active in response to tactile pleasant skin strokes (i.e., caress-like). These studies replicate findings regarding the dual role of perceptual networks in social cognition and draw attention to issues that are currently debated in neurobiological models of empathy and mentalization (Keysers et al., 2010).

The second group of papers investigated neural responses associated with individual differences in attachment. This section begins with a review paper by Gander and Buchheim that describes infant and adult attachment group differences in physiological responsiveness, such as adrenocortical activity, heart rate and skin conductance, and frontal electroencephalographic (EEG) asymmetry. The authors demonstrate the role of secure attachment, as compared with insecure attachment, as a physiological reactivity buffer to stress responses, noting also that investigations examining the most extreme forms of insecurity (disorganized and unresolved attachment) are still lacking. With regard to insecure adult attachment, Wichmann et al. demonstrated using a Reaction Time paradigm that statements derived from insecure AAP responses (typically describing unpleasant, unsatisfying, or conflictual themes) required significantly greater “unconscious” processing time as compared with sentences derived from secure responses.

Several studies specifically investigated the footprint of so called insecure dismissing attachment, the insecure attachment group characterized by regulation strategies that transform or divert conscious attention away from (i.e., avoid) conflictual attachment experience and affect. Kungl et al. studied the neural substrates of emotion regulation by assessing state and trait dependent EEG asymmetries in healthy adolescents judged dismissing on the AAI. The results showed elevated right-frontal brain activity and reduced right parietal brain activity, validating on the neural level the tendencies of these individuals for avoidance/redirection strategies. The ERP findings from the same study group by Leyh et al. confirmed the association of dismissing attachment with insufficient emotion regulation strategies as evidenced by reduced P3 amplitudes presented in a negative emotional context. Krause et al. applied an fMRI approach from the burgeoning field of resting connectivity using an auditing paradigm (excerpts from AAI narratives) to assess the association between a previously described social aversion network and dismissing attachment. These studies taken together suggest that avoidant strategies may be the result of recruitment of neural substrates associated with social withdrawal or dysfunctional emotion regulation.

A long-standing and important debate in the attachment field concerns demonstrated inconsistencies between self-report and narrative interview adult attachment assessment measures. Using fMRI, Yaseen et al. confirmed network pattern outcome differences associated with these two measurement types. Individual differences in scores from a self-report measure (Relationship Scales Questionnaire) were preferentially associated with changes in the activity of dorsal, cognitive/executive function-related networks while individual differences assessed through an interview assessment AAI were associated with modulation of activity of the antagonist “default system” network (Buckner and Carroll, 2007). This finding also suggests that different dimensions of attachment may associated with different emotion regulation strategies. Schneider-Hassloff et al. used electrophysiological approach to assess emotion regulation functioning (associated in other studies with the dorsal network) in relation to mother-child interaction patterns. They report evidence for a neurobiological signature of these patterns in a response inhibition task. The developmental lens adopted by these researchers (as compared with the personality perspective) was important in these studies. They sought evidence for the influence of adaptive emotion regulation strategies, thought to originate in early development, as characterized by effective and balanced recruitment of cognitive processes for top-down control, a central issue in the clinical neurosciences of affect (Ochsner and Gross, 2005; Messina et al., 2016). This developmental perspective is also central to the study by Zimmermann and Spangler. These authors investigated the role of genetic predisposition in modulating emotion regulation and attachment patterns of adolescents using the Late Childhood Attachment Interview (LCAI). Their results showed an interaction between the participants' attachment pattern with mother and a polymorphism of the serotonin transporter promoter region (5-HTTLPR), which has been shown in previous studies to modulate response to early adversity (Canli and Lesch, 2007).

A third group of studies importantly included participants from patient groups, acknowledging that adverse attachment experiences such as maltreatment, loss, and separation have long been known to have enduring consequences on human mental health. These studies addressed the issue of whether neural correlates of differing attachment patterns can shed light on psychopathology using the AAP (Krause et al.; Buchheim et al.; Jobst et al.). Krause et al. reported a significant increase of the neuropeptide oxytocin (OT—the “hormone of affiliation)” after administering the AAP to lactating mothers in a subclinical group. Although plasma OT was independent of the mothers' attachment representations, the finding that secure mothers showed a decrease of cortisol release after the AAP confirms the buffering effect of attachment security on a neuroendocrine level.

The neural patterns associated with attachment in an fMRI study with Borderline Personality Disorder (BPD) were examined using a paradigm that instructed participants to tell AAP stories in the scanner. The results showed significant differences between patients and control. Buchheim et al. found that unresolved attachment in both patients and controls demonstrated enhanced amygdala activation, but only the controls showed frontal activations (DLPFC, RCZ) and top down control. This finding points to possible neural mechanisms in BPD patients with unresolved attachment trauma (the majority attachment pattern associated with BPD in the literature) and their inability to regulate attachment distress. This finding was confirmed also in an OT study by Jobst et al. who demonstrated that only BPD patients with unresolved attachment (assessed with the AAP) showed lower OT in plasma over the course of an exclusion paradigm (cyberball), which again emphasizes the putative mechanisms underlying patients' interpersonal dysregulation.

The final paper reviews attachment, neurobiology and psychosis. Debbané et al. proposed a sophisticated model illustrating five neurobiological pathways through which attachment adversity may augment risk for psychosis.

We invited authors for this Research Topic in Frontiers in Human Neuroscience to submit original research or reviews that addressed topics in the neurobiological domain related to any aspect of attachment that would highlight promising avenues for basic research in developmental psychopathology or the translation of attachment studies into the clinical setting. The authors were using different methodological approaches to respond to this topic. As a result, we achieved an exciting interdisciplinary synthesis of existing knowledge and new perspectives on the human neuroscience of attachment that demonstrates the tremendous development in this field from the seminal first works by Hofer (1994) and Insel and Young (2001). These findings regarding the neural substrates of attachment in healthy individuals lay the foundation of future studies to address a wider range of clinical groups than reported here and the transgenerational transmission of attachment in low and high-risk groups. As a next step, we would like to encourage attachment researchers to evaluate the effectiveness of preventive programs and established interventions (Buchheim et al., 2012) with neurobiological or genetic approaches.

## Author contributions

All authors (AB, CG, HG, RV) have contributed to this Editorial. AB and RV have drafted the Editorial, CG and HG provided intellectual contributions in commenting and revising the manuscript. AB edited its final version.

### Conflict of interest statement

The authors declare that the research was conducted in the absence of any commercial or financial relationships that could be construed as a potential conflict of interest.

## References

[B1] Buchheim A. Viviani R. Kessler H. Kächele H. Cierpka M. Roth G.. (2012). Changes in prefrontal-limbic function in major depression after 15 months of long-term psychotherapy. PLoS ONE 7:e33745. 10.1371/journal.pone.0033745 22470470 PMC3314671

[B2] Buckner R. L. Carroll D. C. (2007). Self-projection and the brain. Trends Cogn. Sci. 11, 49–57. 10.1016/j.tics.2006.11.004 17188554

[B3] Canli T. Lesch K. P. (2007). Long story short: the serotonin transporter in emotion regulation and social cognition. Nat. Neurosci. 10, 1103–1109. 10.1038/nn1964 17726476

[B4] George C. West M. L. (2012). The Adult Attachment Projective Picture System. Attachment Theory and Assessment in Adults. New York, NY: The Guilford Press.

[B5] Hofer M. A. (1994). Hidden regulators in attachment, separation, and loss. Monogr. Soc. Res. Child Dev. 59, 192–207. 10.2307/1166146 7984161

[B6] Insel T. R. Young L. J. (2001). The neurobiology of attachment. Nat. Rev. Neurosci. 2, 129–136. 10.1038/35053579 11252992

[B7] Keysers C. Kaas J. H. Gazzola V. (2010). Somatosensation in social perception. Nat. Rev. Neurosci. 11, 417–428. 10.1038/nrn2833 20445542

[B8] Messina I. Sambin M. Beschoner P. Viviani R. (2016). Changing views of emotion regulation and neurobiological models of the mechanism of action of psychotherapy. Cogn. Aff. Behav. Neurosci. 16, 571–587. 10.3758/s13415-016-0440-5 27351671

[B9] Ochsner K. N. Gross J. J. (2005). The cognitive control of emotion. Trends Cogn. Sci. 9, 242–249. 10.1016/j.tics.2005.03.010 15866151

